# Responding to the Challenge of the Dual COVID-19 and Ebola Epidemics in the Democratic Republic of Congo—Priorities for Achieving Control

**DOI:** 10.4269/ajtmh.20-0642

**Published:** 2020-06-19

**Authors:** Jean B. Nachega, Placide Mbala-Kingebeni, John Otshudiema, Linda M. Mobula, Wolfgang Preiser, Oscar Kallay, Susan Michaels-Strasser, Joel G. Breman, Anne W. Rimoin, Justus Nsio, Steve Ahuka-Mundeke, Alimuddin Zumla, Jean-Jacques Muyembe Tam-Fum

**Affiliations:** 1 Department of Medicine, Centre for Infectious Diseases, Faculty of Medicine and Health Sciences, Stellenbosch University, Cape Town, South Africa;; 2 Department of Epidemiology and International Health, Johns Hopkins Bloomberg School of Public Health, Baltimore, Maryland;; 3 Department of Epidemiology, Infectious Diseases and Microbiology, Center for Global Health, University of Pittsburgh, Pittsburgh, Pennsylvania;; 4 Department of Medical Microbiology and Virology, National Institute of Biomedical Research (INRB), Faculty of Medicine, University of Kinshasa, Kinshasa, Democratic Republic of the Congo;; 5 Health Emergencies Program, COVID-19 Response, Epidemiological Surveillance Team, World Health Organisation, Kinshasa, Democratic Republic of the Congo;; 6 Ebola Response Team, Health, Nutrition and Population, Global Practice, World Bank Group, Washington, District of Columbia;; 7 Division of Medical Virology, Department of Pathology, Faculty of Medicine and Health Sciences, Stellenbosch University, Cape Town, South Africa;; 8 National Health Laboratory Service (NHLS), Cape Town, South Africa;; 9 Erasme Hospital, Université Libre de Bruxelles, Brussels, Belgium;; 10 Columbia University Mailman School of Public Health, New York, New York;; 11 American Society of Tropical Medicine and Hygiene and United States National Institutes of Health, Fogarty International Center, Bethesda, Maryland;; 12 Department of Epidemiology, Fielding School of Public Health, University of California, Los Angeles (UCLA), Los Angeles, California;; 13 Division of Infection and Immunity, Department of Infection, Centre for Clinical Microbiology, University College London, London, United Kingdom;; 14 National Institute for Health Research Biomedical Research Centre, University College London Hospitals, London, United Kingdom

## Abstract

As of June 11, 2020, the Democratic Republic of the Congo (DRC) has reported 4,258 COVID-19 cases with 90 deaths. With other African countries, the DRC faces the challenge of striking a balance between easing public health lockdown measures to curtail the spread of SARS-CoV-2 and minimizing both economic hardships for large sectors of the population and negative impacts on health services for other infectious and noninfectious diseases. The DRC recently controlled its tenth Ebola virus disease (EVD) outbreak, but COVID-19 and a new EVD outbreak beginning on June 1, 2020 in the northwest Équateur Province have added an additional burden to health services. Although the epidemiology and transmission of EVD and COVID-19 differ, leveraging the public health infrastructures and experiences from coordinating the EVD response to guide the public health response to COVID-19 is critical. Building on the DRC’s 40 years of experience with 10 previous EVD outbreaks, we highlight the DRC’s multi-sectoral public health approach to COVID-19, which includes community-based screening, testing, contact-tracing, risk communication, community engagement, and case management. We also highlight remaining challenges and discuss the way forward for achieving control of both COVID-19 and EVD in the DRC.

## INTRODUCTION

The spread of COVID-19 compounds the burden on health services in African countries that have experienced recurrent outbreaks of deadly zoonotic diseases in recent years. As of June 8, 2020, the WHO Africa Region has reported 135,412 COVID-19 cases, with 3,236 deaths from 45 countries.^1^ Most African countries are facing difficult decisions as they attempt to balance efforts to limit the spread of SARS-CoV-2, control local outbreaks of other infectious diseases, and lessen economic hardships and food insecurity for large sectors of the population.^2–4^ The Democratic Republic of the Congo (DRC) recently experienced its tenth Ebola virus disease (EVD) outbreak, the second largest globally after the 2014–2016 West African epidemic, which was recently brought under control. The lessons learned, coordination mechanisms developed, and public health infrastructures put in place for EVD are guiding the public health response to COVID-19 in the DRC, although the two diseases are fundamentally different.^5^ Building on four decades of experience with EVD, we discuss the DRC’s response to COVID-19 and associated challenges, priorities, and innovations for disease control.

## EPIDEMIOLOGICAL SNAPSHOT

**Figure 1.**
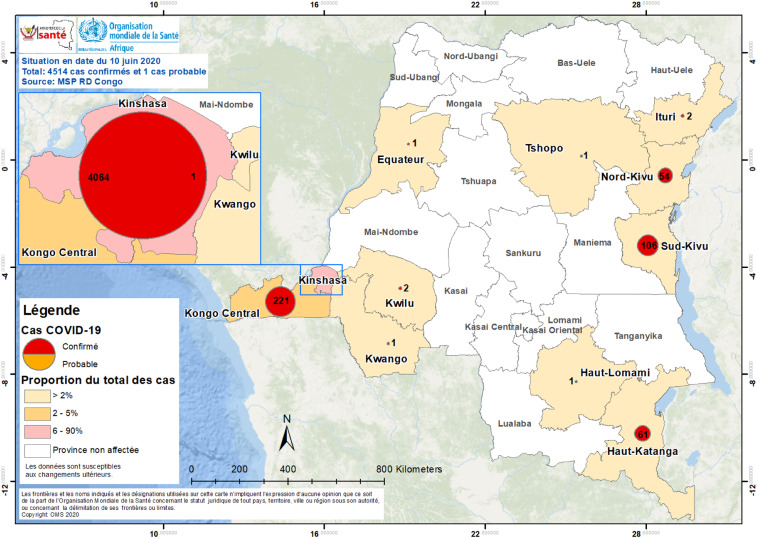
Epidemiology status of COVID-19 in the Democratic Republic of the Congo (as of June 14, 2020).

**Figure 2.**
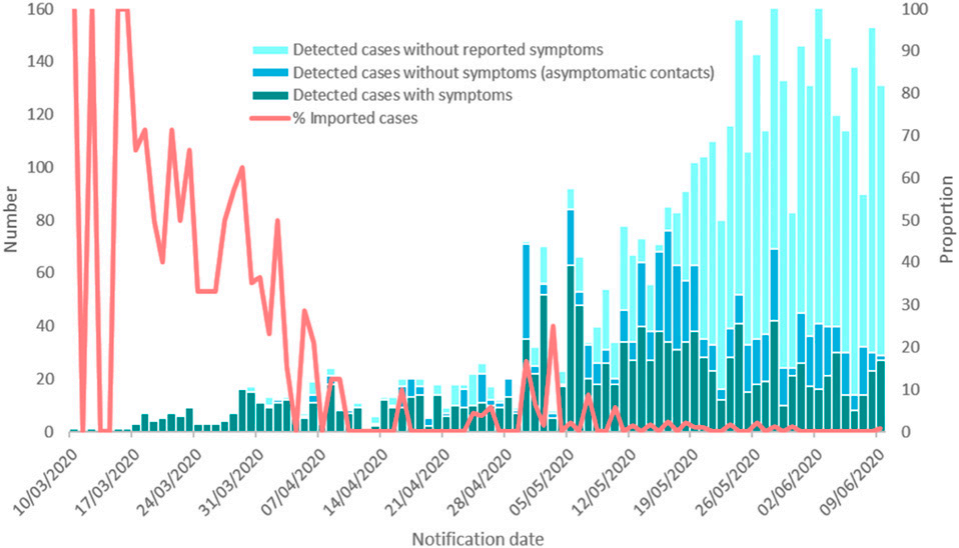
COVID-19 daily case numbers in the Democratic Republic of the Congo (March 10, 2020–June 9, 2020).

Early COVID-19 cases in Africa were mostly due to air travel of infected individuals from Europe.^2,3^ The Democratic Republic of the Congo confirmed its first case of COVID-19 on March 10, 2020. Two days after returning from France, an adult male with cough and fever tested positive in the capital city of Kinshasa. The subsequent early index cases in Kinshasa also occurred among young affluent adult travelers from Europe.^3^ The DRC declared a state of emergency that included travel bans on March 24, and on April 6, a lockdown of the initial COVID-19 hotspot, Gombe, an affluent health zone in Kinshasa, and other selected regions of the country, was instituted. Since then, the number has increased to 4,258 COVID-19 cases, with 90 deaths (case fatality rate of 2.1%) as of June 11, 2020.^1^ To date, the disease has spread to 11 provinces and 54 (10% of total) health zones in the DRC (Figures 1 and 2). As in other African countries, the travel bans and lockdowns have had negative socioeconomic impacts on the population, most of whom live below the poverty line.

## THE DEMOCRATIC REPUBLIC OF THE CONGO’S COVID-19 MULTI-SECTORAL NATIONAL RESPONSE COMMITTEE

A multi-sectoral national committee to organize the COVID-19 response was created following the diagnosis of the first confirmed cases (Figure 2) using lessons learned from the tenth EVD outbreak. The committee, which includes a Presidential Task Force that liaises with the President’s Office and a Strategic and Operational Management Task Force comparable to that of the Ebola Incident Management System, has support from the WHO, U.S. and Africa CDC, World Bank, and U.K. Department For International Development as part of the fourth Strategic Response Plan. The committee’s secretariat is made up of five sections with distinct responsibilities (Supplemental Figure 1). Similar organizations have been set up for the management of the response in the different provinces under the coordination of each governor and provincial minister of health. The DRC government’s COVID-19 task response structure was incorporated into existing health system structures for HIV, tuberculosis, malaria, and other noncommunicable diseases.

## THE COLLISION OF EBOLA VIRUS DISEASE AND COVID-19

The tenth EVD outbreak in the DRC was announced by the Ministry of Health on August 1, 2018, ten days after the ninth outbreak was declared over. Since June 2018, approximately 300,000 people in EVD-affected health zones have been immunized with the Merck rVSV-EBOV vaccine and another 20,000 in Goma with the Janssen Ad26.ZEBOV vaccine.^6^ In addition, investigational drugs were provided to nearly all patients confirmed to have EVD either through the Monitored Emergency Use of Unregistered Investigational Drugs protocol (allowing patients to receive investigational drugs under compassionate use) or as part of the Pamoja Tulinde Maisha (PALM [“Together Save Lives” in Kiswahili]) randomized controlled trial.^7^ A multi-sectoral response, including standard public health measures (surveillance, contact-tracing, active case finding, infection prevention and control, risk communication, community engagement, and safe burials) coupled with community-based interventions such as cash-for-work and water and sanitation hygiene projects, as well as security, eventually controlled a complex outbreak that lasted almost 2 years. Unfortunately, hope held by health officials to declare the outbreak over on April 12, 2020 vanished as a new case was confirmed on April 10, thereby resetting the countdown clock. This was the first time an Ebola outbreak occurred in a conflict zone with an ongoing humanitarian emergency. Numerous factors contributed to the 2-year-long EVD outbreak in the eastern DRC, including a fragile and fragmented health system, population displacement, movement of contacts, disenfranchisement of the community, mistrust, and ongoing armed conflicts.^8^ The DRC continues to face the challenge of having back-to-back EVD outbreaks with limited funding for existing needs.

On June 1, 2020, the DRC government announced an eleventh EVD outbreak occurring in the northwest Équateur Province. The DRC Ministry of Health, in close collaboration with WHO teams who were already on the ground in Mbandaka as part of capacity building, deployed additional multidisciplinary rapid response teams from Goma and Kinshasa to support local teams. According to the WHO, as of June 9, 2020, a total of 12 EVD cases (nine confirmed and three probable) including nine deaths (case fatality rate 75%) were reported in three affected health zones (Wangata, Mbandaka, and Bikoro). Overall, 85.3% (521/611) of contacts were traced, but none turned out to be a suspected EVD case. Also, 1,495 people, including 436 frontline health professionals and close contacts, were vaccinated using the rVSV-ZEBOV-GP vaccine since the beginning of this outbreak.

## LESSONS LEARNED FROM THE EBOLA EXPERIENCE AND HOW THEY ARE BEING APPLIED TO THE COVID-19 RESPONSE

The inability to act rapidly and diagnose and isolate cases of EVD was an important factor in the large-scale progression of the 2014–2016 Ebola outbreak in West Africa. A range of novel Ebola diagnostic tools were trialed and introduced, including automated PCR machines and rapid test kits for point-of-care diagnosis. System-wide support was put in place for safely transporting samples, sourcing reagents, disposal of hazardous materials, and rapid feedback of diagnostic data into public health and clinical decision-making. Although the global COVID-19 pandemic presents unique challenges, several lessons from the EVD outbreaks are informing the COVID-19 response.^6^ First, the Ebola standard operating procedures (SOPs) have been used as a starting point to speed the development and updating of COVID-19 SOPs. Second, Ebola contact follow-up approaches have been leveraged for the follow-up of COVID-19 contacts, with the difference that in this case the duration of follow-up is 14 days, compared with 21 days for EVD. Because persons with COVID-19 may be asymptomatic, contact-tracing includes the collection of respiratory samples on days 7 and 12 from all high-risk contacts of a confirmed case, regardless of symptoms. Third, the follow-up of COVID-19 contacts is modeled after our EVD experience using contact-tracers and community health workers (CHCWs) at the peripheral level: health areas, neighborhoods, and villages. Fourth, the EVD response established mobile laboratories in target provinces. Currently, the COVID-19 response is planning to setup such provincial laboratories for point-of-care (PoC, e.g., GeneXpert) COVID-19 testing. Finally, based on the critical importance of community engagement and feedback during the ninth and tenth EVD outbreaks in the DRC, a mechanism to collect feedback from communities was put in place from the beginning of the COVID-19 response.

## COMMUNITY-BASED COVID-19 SCREENING, TESTING, AND CONTACT-TRACING

As of May 27, 2020, there were 6,389 contacts of the 2,659 confirmed cases traced, resulting in a daily contact-tracing proportion of 92% (Table 1). Among reported confirmed cases, a total of 1,176 (44.2%) were symptomatic (Figure 2).^7,8^ Various organizations involved in the COVID-19 fight across the country, supported by the U.S. Health Resources and Services Administration, are implementing multidisciplinary teams of physicians, nurses, midwives, pharmacists, medical students, and CHCWs for COVID-19 sensitization, screening, and testing activities endorsed by the Ministry of Health, the community, and faith leaders. When a suspect case of COVID-19 is identified based on the presence of signs or symptoms, epidemiological links, or being a high-risk contact, CHCWs send a COVID-19 alert notification to the emergency operations center, and a surveillance team is deployed to investigate. When the suspected case meets the case definition for COVID-19, a respiratory sample is taken, and the person is quarantined at home or in a designated COVID-19 isolation unit while awaiting laboratory results. If the test result is positive, then the patient is referred to the case management team to initiate care.

**Table 1:** COVID-19 contact-tracing in affected provinces in the Democratic Republic of the Congo (March 10, 2020–May 27, 2020)

Province	Total health zones	Affected health zones		Total confirmed cases	Total contacts	Contacts traced	
		*N*	%			*N*	%
Kinshasa	35	34	97	2,394	4,769	4,402	92
North Kivu	34	4	12	35	523	401	77
Higher Katanga	27	5	19	21	337	337	100
Kongo Central	31	5	16	189	760	742	98
South Kivu	34	2	6	16	0	0	Na
Kwilu	34	2	6	2	0	0	Na
Ituri	36	2	6	2	0	0	Na
Democratic Republic of the Congo—nationwide	518	54	10	2,659	6,389	5,882	92

NA = not applicable.

Globally, the current gold standard test for the diagnosis of SARS-CoV-2 infection is detection of viral RNA in a sample from the respiratory tract by RT-PCR.^9–11^ Laboratories with skilled staff and the RT-PCR equipment to perform these tests are scarce in the DRC (Table 2); all COVID-19 testing is performed at the National Institute of Biomedical Research in Kinshasa, a national referral laboratory. Because of sample transport from the provinces, turnaround times are lengthy for samples collected from provinces, causing delays in diagnoses. Of note, PoC or near-patient solutions would be preferable.^12,13^ The GeneXpert platform, already in place for TB testing across Africa, is an attractive option, but drawbacks include cost and limited supplies of SARS-CoV-2 cartridges. PoC viral antigen detection is not yet sufficiently sensitive.^14^ Serological testing for antiviral antibodies is unavailable in the DRC and is unsuitable for diagnosing active COVID-19 cases.^15^

**Table 2:** Challenges and priority solutions for optimizing COVID-19 response in the Democratic Republic of the Congo

		Early and late challenges	Priority solutions
1	Social distancing, barrier measures, and handwashing	Some community members do not believe that disease exists	Scale-up community COVID-19 sensitization and barrier measure messages involving community leaders, role models in music and sports, traditional leaders, etc.
		Poverty levels limit respect for the application of barrier measures/socio-distancing, and handwashing	Scale-up distribution of sanitizers and locally-made masks to communities supported by government and multilateral donors and partners (e.g., the World Bank)
		Running water is scarce in some communities	
2	SARS-CoV-2 testing	One laboratory at the national level, the INRB in Kinshasa, performs all the COVID-19 RT-PCR testing	Build capacity for RT-PCR SARS-CoV-2 testing at referral laboratories in provinces
		Consequences: Late detection and delays in the delivery of results to provinces	Acquisition and decentralization of screening and PCR testing using PoC machines in provinces
		Shortage of PoC machines (e.g., GeneXpert) and reagents/cartridges	Leverage infrastructure, human resources, medical management and training platform of Ebola Viral Disease for COVID-19
			Increase resources in affected provinces and adequate preparedness for provinces that are not yet affected by the virus
3	Case management	Insufficient medical and personal protective equipment	Increase logistical support (e.g., protective equipment, medical equipment, medicines, and means of transportation)
		Limited technical capacity of COVID-19 case management among healthcare providers	Leverage infrastructure, human resources, medical management, and training platform of Ebola viral disease for COVID-19
		Limited logistical resources to carry out response activities in the province’s remote areas	Increased numbers of multidisciplinary healthcare workers and trained CHWs
			Ensure continuity of care for NCDs and other comorbidities

CHCWs = community healthcare workers; DRC = Democratic Republic of the Congo; PPE = personal protection equipment; POC = point of care; RT-PCR = reverse transcriptase–PCR.

## CASE MANAGEMENT

Treatment of moderate and severe cases of COVID-19 requires hospitalization for supportive care, oxygen, and anticoagulation as per WHO guidelines.^16^ Remdesivir, which has been shown to be effective in reducing the length of hospitalization for moderately severe cases, is not yet available in the DRC.^17^ Weak health systems in the DRC, with limited intensive care beds, oxygen supply, ventilators, and trained staff, remain a key challenge in the management of COVID-19, especially as case numbers rise. Several hospitals were identified as reference centers for the treatment of COVID-19 as part of the National Plan. Furthermore, building on an existing innovative tele-mentoring program developed to capacitate nurses and other frontline healthcare workers, a series of in-service COVID-19 training modules covering triage, infection prevention and control, testing, maintenance of essential services, and other topics was developed. At the start of the outbreak, only 60 ventilators were available country-wide and oxygen supplies were limited, and there was minimal technical capacity to provide intensive care. A clinical protocol was developed by the case management commission with support from technical partners. Bilateral and multilateral partnerships are scaling-up donations including medical and personal protective equipment to the reference hospitals, and training has been provided to clinical staff to ensure optimal care and prevention of infection of healthcare workers.^18^ Remaining challenges and priority solutions are listed in Table 2.

## CONCLUSIONS AND WAY FORWARD

As the DRC decides how best to control the COVID-19 pandemic, it is essential to reflect on lessons learned from past and current EVD outbreaks. The DRC must adapt the available infrastructure and protocols to COVID-19 while embedding community needs and concerns into its response. The country must also significantly invest in its fragile health systems to ensure equity, stability, and global health security. Control of the COVID-19 pandemic in the DRC will be possible only with efficient community screening, testing, and contact-tracing as well as behavioral modification, all of which require adequate local and national resources and enough trained and protected personnel. By addressing the challenges, the DRC and other countries in Africa can limit the impact of the COVID-19 pandemic on the health of its already vulnerable citizens.

## Supplemental figure

Supplemental materials
