# Supplementary material for: Responding to the Challenge of the Dual COVID-19 and Ebola Epidemics in the Democratic Republic of Congo—Priorities for Achieving Control
Source: Am J Trop Med Hyg. 2020 Jun 19;103(2):597–602. doi: 10.4269/ajtmh.20-0642 (PMC7410434; doi:10.4269/ajtmh.20-0642)
Supplement: Supplementary file 1 [file tpmd200642.SD1.docx]

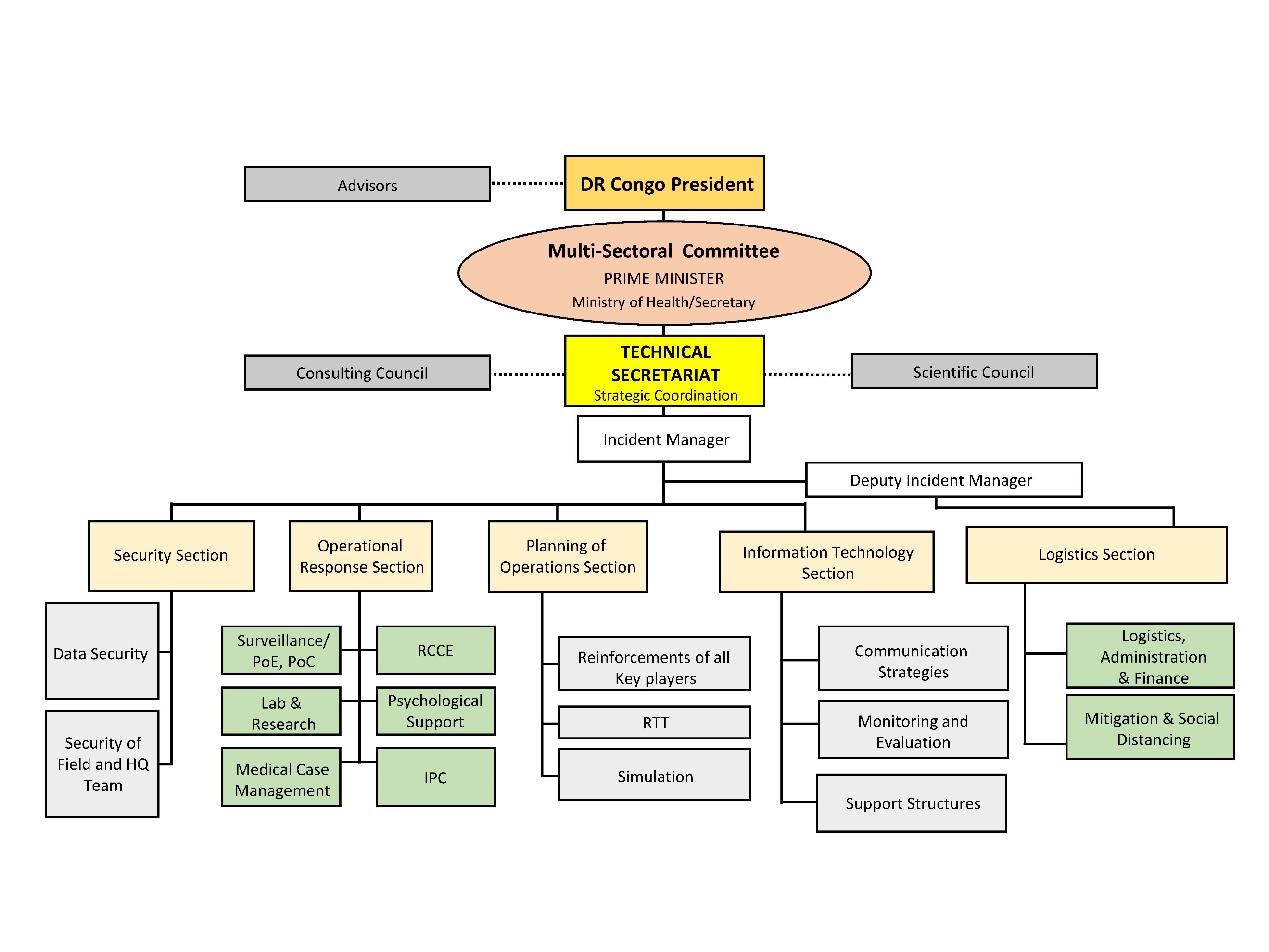


**Supplemental e-Figure 1. The Democratic Republic of Congo multisectoral committee for the response to COVID-19**
